# ^225^Ac-Labeled Somatostatin Analogs in the Management of Neuroendocrine Tumors: From Radiochemistry to Clinic

**DOI:** 10.3390/pharmaceutics15041051

**Published:** 2023-03-24

**Authors:** Léa Rubira, Emmanuel Deshayes, Lore Santoro, Pierre Olivier Kotzki, Cyril Fersing

**Affiliations:** 1 Nuclear Medicine Department, Institut Régional du Cancer de Montpellier (ICM), University Montpellier, 34090 Montpellier, France; 2 Institut de Recherche en Cancérologie de Montpellier (IRCM), INSERM U1194, University Montpellier, Institut Régional du Cancer de Montpellier (ICM), 34298 Montpellier, France; 3 IBMM, University Montpellier, CNRS, ENSCM, 34293 Montpellier, France

**Keywords:** actinium-225, radionuclide production, radiolabeling, targeted radionuclide therapy, targeted alpha-therapy, radiobiology, neuroendocrine tumors, ^225^Ac–DOTATATE, radiopharmaceuticals

## Abstract

The widespread use of peptide receptor radionuclide therapy (PRRT) represents a major therapeutic breakthrough in nuclear medicine, particularly since the introduction of ^177^Lu-radiolabeled somatostatin analogs. These radiopharmaceuticals have especially improved progression-free survival and quality of life in patients with inoperable metastatic gastroenteropancreatic neuroendocrine tumors expressing somatostatin receptors. In the case of aggressive or resistant disease, the use of somatostatin derivatives radiolabeled with an alpha-emitter could provide a promising alternative. Among the currently available alpha-emitting radioelements, actinium-225 has emerged as the most suitable candidate, especially regarding its physical and radiochemical properties. Nevertheless, preclinical and clinical studies on these radiopharmaceuticals are still few and heterogeneous, despite the growing momentum for their future use on a larger scale. In this context, this report provides a comprehensive and extensive overview of the development of ^225^Ac-labeled somatostatin analogs; particular emphasis is placed on the challenges associated with the production of ^225^Ac, its physical and radiochemical properties, as well as the place of ^225^Ac–DOTATOC and ^225^Ac–DOTATATE in the management of patients with advanced metastatic neuroendocrine tumors.

## 1. Introduction

### 1.1. About Neuroendocrine Tumors

Neuroendocrine tumors (NETs) form a heterogeneous group of malignancies with a wide variety of histology and nomenclature. The term “neuroendocrine” is used to describe cells that are widely spread throughout the body, with both neurological and endocrine characteristics [1]. Neurological properties are based on the presence of dense granules similar to those found in serotonergic neurons that store monoamines [2]; endocrine properties refer to the synthesis and secretion of such mediators [3]. Thus, this broad definition includes neoplasms occurring in nerve structures (e.g., ganglia and paraganglia), in straight endocrine organs (e.g., pituitary gland, thyroid, parathyroid or adrenal) and in the diffuse neuroendocrine system of various organs.

NETs account for approximately 0.5% of all newly diagnosed malignancies [4], with increasing incidence over the years [5]. As an example, the age-adjusted NET incidence in the UK increased 3.7-fold between 1995 and 2018, from 2.35 to 8.61 per 100,000 [6]. A similar significant increase over time has been reported in other geographical areas [7,8,9]. However, epidemiology data on NETs are difficult both to collect and to interpret because of the heterogeneity of their classification, the different methods of patient identification, and the lack of large population databases in most countries. Furthermore, the distribution of NETs according to the primary site slightly varies in the different geographical areas studied, which could reflect ethnic or genetic specificities [10,11]. Overall, the most common primary sites are the gastrointestinal tract (about two in three patients) and the lung (about one in four patients). Between one and two out of ten patients are metastatic at the time of diagnosis [8,12].

### 1.2. Somatostatin Receptors and Octreotide Analogs

**Figure 1:**
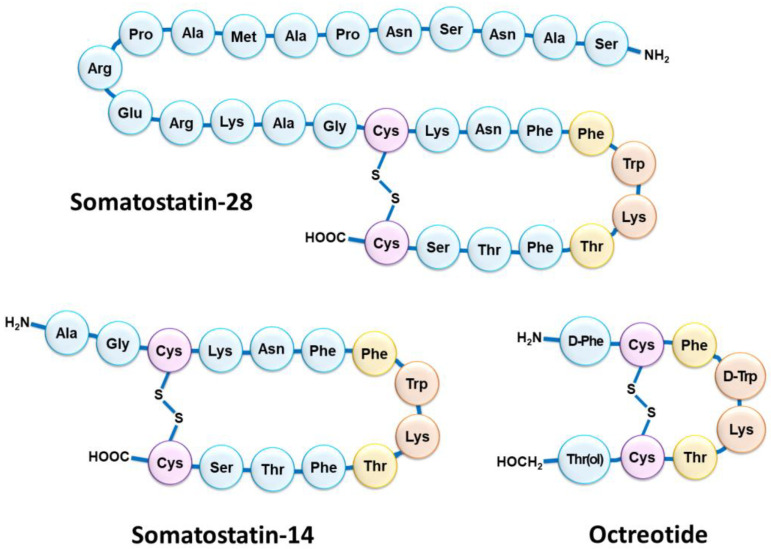
Schematic structure of the two natural isoforms of somatostatin (SS-14 and SS-28) and octreotide. The amino acid residues Cys (purple) form an intramolecular disulfide bridge; the amino acid residues Trp and Lys (orange) included in a β-turn are necessary for biological activity; the nearby amino acid residues Phe and Thr (yellow) are in favor of good biological activity but accept slight modulation.

Although NETs are heterogeneous diseases in their pathophysiology and clinical expression, they usually share the characteristic of overexpressing somatostatin receptors (SSTRs) [13]. Five SSTR subtypes are described (SSTR1 to SSTR5), SSTR2 being the most frequently encountered in differentiated NETs [14]. However, several subtypes can be expressed concomitantly on tumor cells in various combinations and proportions [15,16]. NETs overexpressing SSTRs most often have a gastrointestinal, pancreatic, bronchial, pulmonary, or even thymic or breast origin. SSTRs belong to the G-protein-coupled receptor family and are localized at the cell membrane. Their natural peptide ligand, somatostatin, is found in humans under two different forms: one of 14 amino acids (SS-14) and one of 28 amino acids (SS-28) (Figure 1) [17,18]. Natural somatostatin has been shown to be unsuitable for in vivo use due to its short plasma half-life (about 3 min) [19]. Analogues of this hormone, more resistant to enzymatic degradation, have therefore been developed by making various modifications to the natural molecule [20,21]. The introduction of D-series amino acids to improve in vivo stability, the retention of the minimum chain length to maintain biological activity, the use of the hexapeptide motif Cys-Phe-D-Trp-Lys-Thr-Cys and the elongation of the N- and C-terminal ends allowed the characterization, in 1982, of the most stable active somatostatin analog known as octreotide (Figure 1) [22].

**Figure 2:**
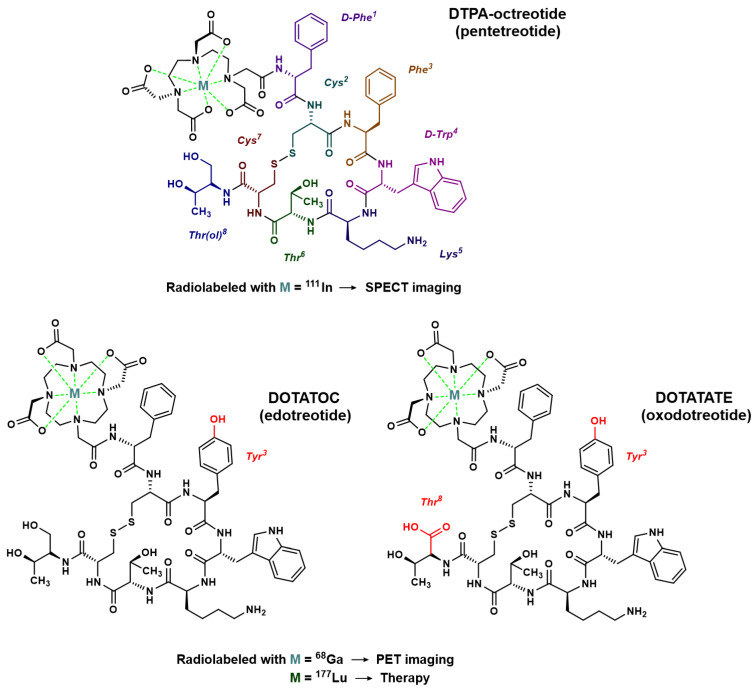
Chemical structure of octreotide analogs most commonly used for molecular imaging or treatment of NETs. In red: modifications of the peptide sequence in comparison to octreotide.

In order to introduce an anchoring site for radiometals, the N-terminal end of octreotide was first functionalized with an acyclic chelator of the diethylenetriamine penta-acetic acid (DTPA) family, able to coordinate ions such as indium in the +3 oxidation state. Thus, since the mid-1990s, the radiopharmaceutical drug [^111^In]In–DTPA–octreotide has been used for scintigraphy imaging of somatostatin receptors [23]. This drug is mainly used in gastroenteropancreatic neuroendocrine tumors and carcinoid tumors [24,25], but also in pituitary secretory tumors, paragangliomas, medullary thyroid carcinomas, pheochromocytomas, meningiomas and Merkel cell tumors [26]. [^111^In]In–DTPA–octreotide binds with moderate affinity to SSTR2; replacement of the Phe^3^ of octreotide by a Tyr^3^ (TOC) leads to improved affinity for SSTR2. In addition, the C-terminal introduction of a Thr^8^ (TATE) in place of the Thr(ol)^8^ of TOC provides a further improvement in affinity for SSTR2 (Figure 2) [27]. Moreover, since DTPA is a poor chelating agent for other radiometals with a +3 oxidation state such as gallium-68, yttrium-90 or lutetium-177, a 1,4,7,10-tetra-azacyclododecane–1,4,7,10-tetra-acetic acid (DOTA) moiety was considered to replace DTPA (Figure 2). This chelator allows the formation of thermodynamically and kinetically stable complexes with a series of radiometals including ^111^In, ^68^Ga, ^90^Y and ^177^Lu. For example, the somatostatin-derived conjugates DOTATOC (edotreotide) and DOTATATE (oxodotreotide) are both currently used as ^68^Ga-radioconjugates for PET imaging and radiolabeled with ^177^Lu for therapy.

### 1.3. SSTR Targeting for Peptide Receptor Radionuclide Therapy

Radionuclide therapy consists of the administration of a vector molecule labeled with a particle-emitting radioelement (either β or α) for therapeutic purposes. This approach is called radiopeptidotherapy or peptide receptor radionuclide therapy (PRRT) in the case of NETs, as the vector molecules used so far have been somatostatin analogs functionalized by a chelating agent [28]. This treatment method is recommended for metastatic or inoperable diseases with a positive expression of SSTR2. In NET radiopeptidotherapy, a first generation of molecules containing the Auger-emitter ^111^In was developed and evaluated [29,30,31,32], followed by a second generation of PRRT agents radiolabeled with the beta-emitter ^90^Y [33,34,35,36]. Subsequently, in the early 2000s, ^177^Lu–[DOTA^0^,Tyr^3^]octreotate emerged as an advantageous alternative, emitting both β- and γ-radiation [37]. The β- particles of ^177^Lu are characterized by a maximum energy E_max_ of 0.5 MeV and a mean energy of 133.3 keV (lower than the ^90^Y E_max_ of 2.28 MeV and E_mean_ of 932.9 keV, respectively, improving the irradiation of small tumors) [38] and an average path in the tissues of 2 mm (also lower than the ^90^Y tissue penetration of 11 mm). This radioelement is characterized by a physical half-life of 6.7 days. Thus, a number of non-randomized, uncontrolled clinical trials were undertaken, especially with [^177^Lu]Lu–DOTATATE. An early cohort study reported efficacy results for [^177^Lu]Lu–DOTATATE in 310 patients with various types of gastroenteropancreatic (GEP) NETs: the overall tumor response rate was 46% and the overall survival (OS) from the start of treatment was 46 months [39]. In 443 patients with GEP, lung or other NETs, Brabander et al. reported a slightly longer median OS (63 months) and a comparable median progression-free survival (PFS) (29 months) [40]. In another prospective phase 2 study involving 52 patients with pancreatic (p) NETs, half of the enrollment received 27.8 GBq of [^177^Lu]Lu–DOTATATE and half received 18.5 GBq [41]. The high-dose cohort showed a complete response in 12%, a partial response in 27%, and stable disease in 46% of the patients, compared with 4%, 15%, and 58% of the patients, respectively, in the low-dose group. Another retrospective analysis of 68 patients with pancreatic NETs showed comparable results [42]. [^177^Lu]Lu–DOTATATE also demonstrated efficacy in small bowel NETs with an overall disease control rate of 91.8% [43]. A study of 265 patients also showed symptomatic improvement following PRRT, observed in 53% to 70% of patients [44]. Numerous other reports have confirmed the clinical benefits of ^177^Lu-PRRT targeting SSTRs, either in pNETs [45,46,47], gastroenteric NETs [48,49] or GEP NETs [50], sometimes associated with other types of diseases (e.g., unknown primary tumor) [51,52,53,54,55]. Notably, particular attention has been paid to the benefit of [^177^Lu]Lu–DOTATATE in the management of lung NETs [56,57]. In two pilot studies in pediatric patients, treatment with [^177^Lu]Lu–DOTATATE even resulted in therapeutic responses in children with refractory neuroblastoma [58,59]. The NETTER-1 study was the first multicenter, randomized, controlled phase 3 trial comparing [^177^Lu]Lu–DOTATATE to octreotide. The study included 229 patients with unresectable metastatic intestinal NETs expressing SSTRs. Patients were randomized to receive either PRRT with octreotide (7.4 GBq of [^177^Lu]Lu–DOTATATE every 8 weeks for four administrations, with 30 mg octreotide every 4 weeks) or octreotide alone (60 mg every 4 weeks). The estimated PFS rate at 20 months was 65.2% in the PRRT group and 10.8% in the control group; the response rate was 18% in the PRRT group versus 3% in the control group [60]. The safety profile of [^177^Lu]Lu–DOTATATE was generally good, as the rates of grade 3 or 4 adverse events were similar in the two groups; however, grade 3 or 4 neutropenia, thrombocytopenia, and lymphopenia were reported in 1%, 2%, and 9% of patients, respectively, in the [^177^Lu]Lu–DOTATATE group versus no patients in the control group. Furthermore, in addition to improving PFS, [^177^Lu]Lu–DOTATATE showed a significant benefit to quality of life in patients with progressive midgut NETs [61], although the final OS did not significantly differ between the two groups [62]. Following the results of the NETTER-1 study, EMA and FDA approved [^177^Lu]Lu–oxodotreotide for the treatment of GEP NETs expressing SSTRs [63,64]. To date, in addition to several phase 1 and 2 clinical trials, the main prospective randomized phase 3 clinical trial currently ongoing in high-grade 2 and 3 NETs is COMPOSE [65]. This study investigates the early use of [^177^Lu]Lu–DOTATOC as a first- or second-line treatment in patients with well-differentiated G2 or G3 GEP NETs versus the standard of care (either everolimus, CAPTEM or FOLFOX). Lastly, the final results of some studies that no longer include patients are currently pending, as is the case for COMPETE (NCT 03049189) [66,67] assessing efficacy and safety of [^177^Lu]Lu–DOTATOC compared to everolimus in patients with GEP NETs; OCLURANDOM (NCT02230176) [68], a phase 2 trial investigating the antitumor efficacy of [^177^Lu]Lu–DOTATATE compared to sunitinib in pancreatic NET; and more recently, the NETTER-2 trial (NCT03972488) studying the efficacy and safety of [^177^Lu]Lu–DOTATATE in patients with grade 2 and grade 3 advanced GEP NETs compared to high-dose long-acting octreotide.

### 1.4. PRRT Using Somatostatin Analogs Radiolabeled with Alpha-Emitters

Although β-PRRT remains an approved treatment for unresectable metastatic NETs, some tumors show resistance to β-emissions despite somatostatin receptor expression [69]. Furthermore, not all treated patients achieve partial or complete response following SSTR-targeting ^177^Lu-PRRT, and relapse is often observed in the years post-treatment [70]. Thus, among the strategies considered in an effort to overcome these drawbacks, octreotide derivatives radiolabeled with alpha-emitting radionuclides have received particular attention [71]. Within this group of radioisotopes, radium-223 (alkaline earth metal, group 2) has been extensively studied both in vitro and in vivo, and has paved the way for the use of alpha-emitting radioelements in patients [72]. To date, radium-223 is used in its dichloride form for the treatment of symptomatic bone metastases in patients with castration-resistant prostate cancer, without known visceral metastatic disease. However, due to its particular chemistry, ^223^Ra is not suitable for DOTA-peptide radiolabeling. Thus, a special interest has emerged for several α-emitting lanthanides (e.g., ^149^Tb) and actinides (e.g., ^227^Th and ^225^Ac), as well as some radioelements from their decay chain (e.g., ^213^Bi) to achieve a convenient complex formation with DOTA [73]. An initial preclinical evaluation of ^213^Bi–DOTATOC showed its potential value in NETs resistant to ^177^Lu-PRRT [74,75,76,77], these properties being promptly confirmed in the clinic [78]. Nevertheless, targeted alpha-therapy (TAT) involving actinium-225 has gained even greater popularity over the last decade, particularly with applications in prostate cancer [79] and neuroendocrine tumors [80].

This review emphasizes TAT with ^225^Ac-containing somatostatin analogs, providing an in-depth summary of ^225^Ac physical, radiobiological and chemical properties, while highlight ^225^Ac production methods. The preclinical development and clinical use of ^225^Ac-labeled peptides targeting SSTRs are discussed in detail, outlining both the advantages and current limitations of this emerging NET treatment option.

## 2. Actinium-225: Decay Characteristics, Radiobiological and Dosimetry Considerations

### 2.1. Physical Properties of Actinium-225

**Figure 3:**
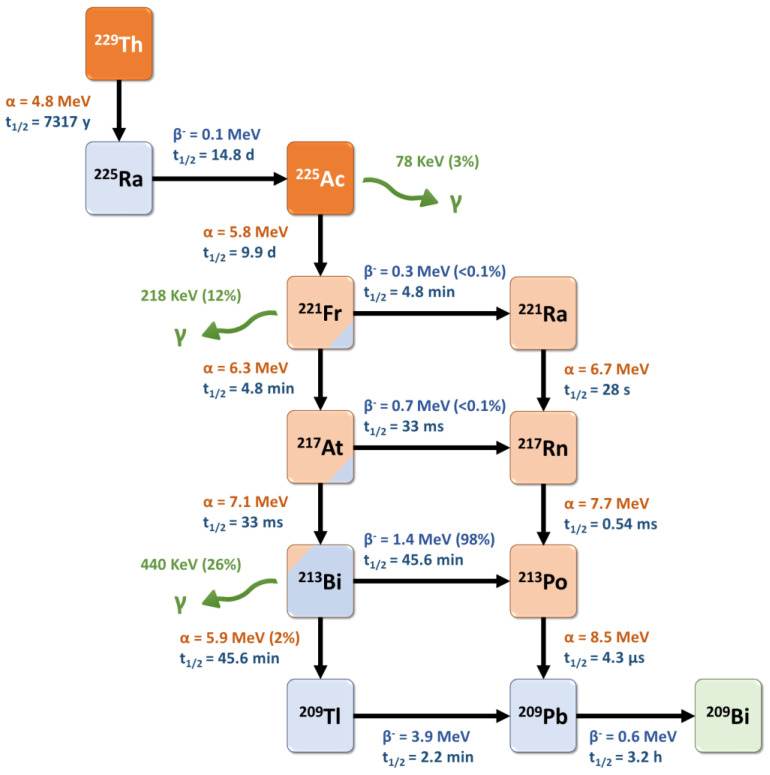
Decay scheme for one type of production and radioactive decay of ^225^Ac.

Actinium-225 is a relatively long-lived pure alpha-emitter, with a half-life of 9.9 days that is well-suited for radionuclide therapy applications and for centralized industrial production, distant from the (pre)clinical user sites. It is formed from the ^229^Th decay product ^225^Ra and decays via a cascade of six short-lived daughter radionuclides to the nearly stable bismuth-209 (Figure 3) [81]. These intermediates include francium-221 (t_1/2_ = 4.8 min, 6.3 MeV α-particle and 218 keV γ-emission), astatine-217 (t_1/2_ = 33 ms, 7.1 MeV α-particle), bismuth-213 (t_1/2_ = 45.6 min, 5.9 MeV α-particle, 1.4 MeV β-particle and 440 keV γ-emission), polonium-213 (t_1/2_ = 4.3 μs, 8.5 MeV α-particle), thallium-209 (t_1/2_ = 2.2 min, 3.9 MeV β-particle) and lead-209 (t_1/2_ = 3.2 h, 0.6 MeV β-particle) before reaching ^209^Bi. Overall, the predominant decay pathway of ^225^Ac produces four alpha-particles with energies ranging from 5.8 to 8.5 MeV and associated tissue ranges of 47 to 85 µm. In addition, the cascade includes two main beta-disintegrations of 1.4 and 0.6 MeV maximum energy. Therefore, ^225^Ac is considered as an in vivo radionuclide generator or a “nanogenerator” with regard to its decay chain.

**Figure 4:**
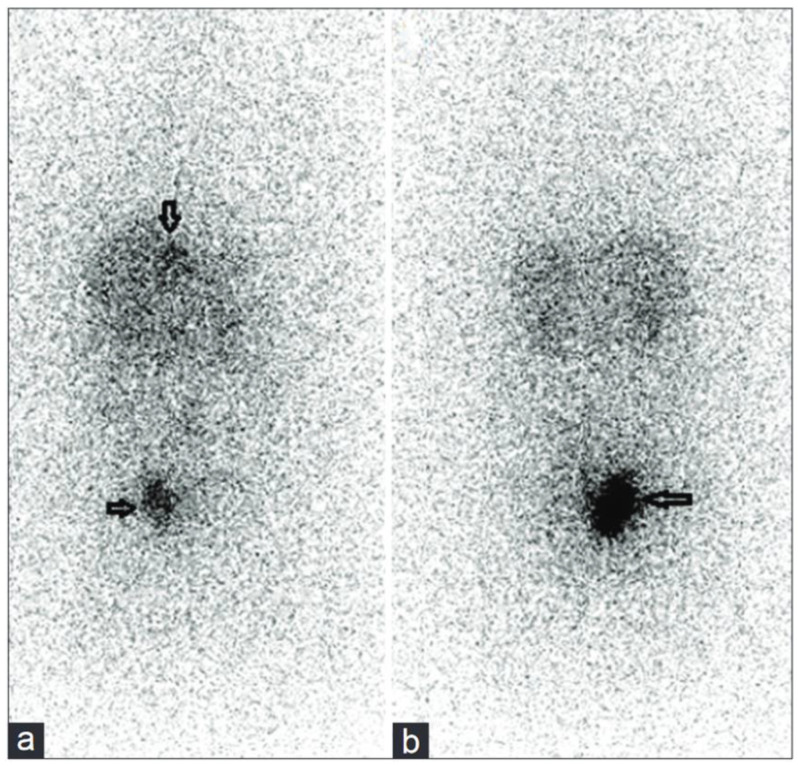
Anterior (**a**) and posterior (**b**) post-therapy whole-body SPECT/CT scans showing intense accumulation in pararectal lesion (horizontal arrows) and liver metastases (vertical arrows) in a patient with a grade 2 NET, 24 h after treatment with 5.5 MBq [^225^Ac]Ac–DOTATATE, acquired for 30 min using 256 × 1024 matrix and high-energy general-purpose collimators (218 keV and 440 keV photon energies with 20% window width), showing increased uptake in pararectal lesions, lymph nodes, and liver metastases [82].

Interestingly, two daughter radionuclides of ^225^Ac (^221^Fr and ^213^Bi) also emit a gamma-photon (218 keV, 11.4% and 440 keV, 25.9%, respectively) which facilitates their tracking after administration (Figure 4). Thus, these gamma-photons can be useful for imaging and dosimetry studies. However, in the early preparation steps of such radiopharmaceuticals, radiolabeling reaction monitoring based on these photons’ detection is quite complex as the secular equilibrium (>6 h) has to be reached before calculating the radiochemical yield. These specific points are further discussed below.

### 2.2. Radiobiological Properties of Actinium-225 

^225^Ac appears as a particularly cytotoxic radionuclide, regarding its long half-life and the multiple alpha-particles generated in its decay chain.

**Figure 5:**
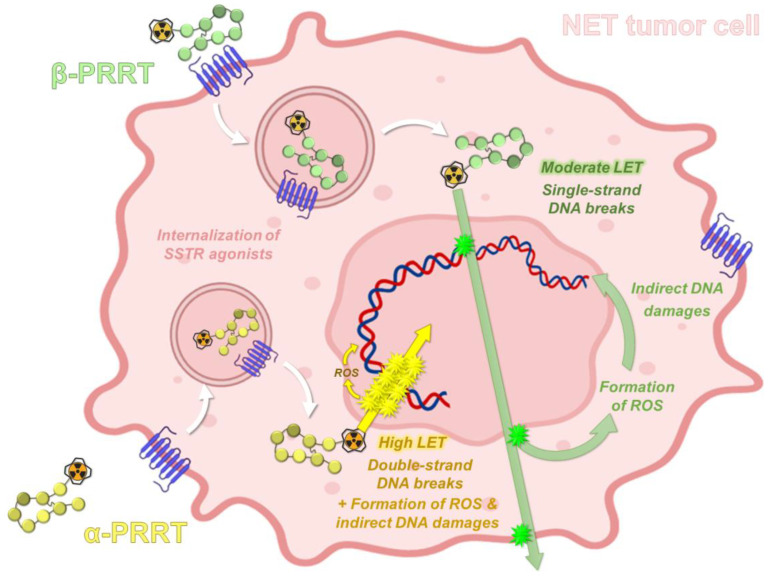
Principle of SSTR-targeting PRRT based on radiolabeled somatostatin analogues. PRRT: peptide receptor radionuclide therapy; SSTR: somatostatin receptors; LET: linear energy transfer; ROS: reactive oxygen species.

Alpha-particles have a shorter range in tissues (<0.1 mm) than beta-particles (around 2 mm for ^177^Lu), which allows the selective killing of targeted cancer cells and theoretically reduces the risk of toxicity to surrounding healthy tissues. In radiation therapy, tumor cell death is directly related to the absorbed doses (i.e., energy deposit, expressed in Grays, with 1 Gy = 1 J/kg) inducing DNA damage that may be direct or indirect (water ionization or excitation generating reactive oxygen species) after interaction with the ionizing particle or radiation. Damage to cell membranes and other cell components, such as mitochondria, may also result in cell death. For the same absorbed dose, the different types of radioactive particles do not have the same biological effects. Alpha-particle emitters have a higher linear energy transfer (LET), which represents the energy deposit by length (or volume), with values around 50–230 keV·µm^−1^ in water [83]. Compared to beta-particle emitters and for the same physical absorbed dose, alpha-particles generate a higher density of ionization and excitation along their track. This causes various types of damage that are more difficult to repair, especially DNA double-strand breaks, explaining the higher relative biological effectiveness (RBE) of alpha-particles (Figure 5). 

These differences between α- and β-PRRT targeting somatostatin receptors were specifically studied by Graf et al. by quantifying the DNA double-strand breaks caused in vitro in AR42J cells by either ^177^Lu- or ^225^Ac-labeled DOTATOC [84]. The median effective dose (ED_50_) was calculated as 14 kBq/mL after 48 h for [^225^Ac]Ac–DOTATOC and 10 MBq/mL (i.e., 714-fold higher) for its ^177^Lu analogue, which is consistent with the differing radiation properties of the two radioisotopes. Interestingly, the amount of double-strand breaks for equitoxic doses of both radioconjugates were comparable. However, a greater tail moment in the comet assay and a higher fraction of polyploid cells suggested more severe cell alterations for high [^225^Ac]Ac–DOTATOC activities. A trend towards late DNA damage with α-PRRT was also highlighted. Additional in vivo experiments with equitoxic doses of the two radiopeptides showed a strong tumor growth delay of 20 days after 40 kBq ^225^Ac and 15 days after 30 MBq ^177^Lu. In relation to these slightly better results obtained with [^225^Ac]Ac–DOTATOC, the authors hypothesized that biological mechanisms such as different DNA repair processes and apoptosis pathways could potentially increase the therapeutic efficacy of α-PRRT. Therefore, alpha-particles may be an interesting option for tumors resistant to β-radiation and conventional therapies [85].

### 2.3. Dosimetry for Targeted Alpha-Therapy with ^225^Ac

The purpose of dosimetry in radionuclide therapy is to understand or predict the likely biological effects, such as toxicity and efficacy, of a radiopharmaceutical drug on a patient. Evaluating the absorbed dose in relevant organs and tumors requires essential parameters including the spatial and temporal biodistribution of the administered radiopharmaceutical (in order to estimate the total number of radionuclide disintegrations in different tissues and tumors, determined by multi-time-point photon imaging) and information about both the physical properties of the radionuclide and the patient anatomy. In the case of alpha-emitter-labeled radiopharmaceuticals, accurate quantitative imaging is particularly challenging due to the low yield of imageable photons emitted, the very low activity administered, the short path length and heterogeneous distribution in tissue, and the multiple daughter radionuclide redistributions. However, biodistribution and dosimetry research involving ^225^Ac has emerged in the last few years using different approaches. These include the direct detection of gamma-emissions by gamma-cameras [86,87], dosimetry based on a surrogate nuclide such as ^177^Lu that can be imaged (particularly for [^225^Ac]Ac–PSMA-617 treatment [88,89]), pharmacokinetic modeling [90], and small-scale and microdosimetry [91,92]. Additionally, preclinical dosimetry studies and animal models are essential in the development of dosimetry research with alpha-particle emitters, specifically for ^225^Ac [93].

## 3. Radiochemical and Preclinical Development of [^225^Ac]Ac–DOTATATE

### 3.1. Production of Actinium-225

Two isotopes of actinium, ^227^Ac and ^228^Ac, exist in nature within the natural decay chain of uranium-235 and thorium-232, respectively [94]. However, neither of these two isotopes is used in the clinic, with ^228^Ac representing a minimal part of natural actinium and ^227^Ac having a very long half-life (t_1/2_ = 21.77 y). Therefore, ^225^Ac is the only one of the more than 30 known actinium isotopes to be used in preclinical and clinical studies to date.

The main method for generating ^225^Ac for clinical use is through radiochemical extraction following the decay of ^229^Th (t_1/2_ = 7397 y), which originates from reactor-bred ^233^U [95,96]. The main sources of ^229^Th in the world for ^225^Ac used in preclinical and clinical studies are Oak Ridge National Laboratory (ORNL, Oak Ridge, TN, USA) [95], the Institute of Physics and Power Engineering (IPPE, Obninsk, Russia) [97], and the Directorate for Nuclear Safety and Security of the Joint Research Center of the European commission (JRC, Halstenbek, Germany), formerly the Institute for Transuranium Elements (ITE, Karlsruhe, Germany) [98]. More recently, the Canadian Nuclear Laboratories set up a ^225^Ac production chain that could supply up to 3.7 GBq of this radioisotope annually [99]. In this latter process, anion-exchange chromatography is used to retain bulk ^229^Th and recover ^225^Ra and ^225^Ac with 8 M nitric acid. Then, dual TEVA/DGA-N cartridges are used to retain breakthrough ^229^Th (TEVA resin) and ^225^Ac (DGA-N cartridge), removing ^223^Ra. After rinsing the DGA-N cartridge with 8 M HCl, ^225^Ac is finally eluted in a minimum volume of 0.05 M HCl, yielding actinium in its AcCl_3_ chemical form. The purification of ^225^Ac represents a crucial step: various alternative separation protocols have therefore been described, based on solid-phase extraction [100,101,102,103], anion or cation exchange [104], or liquid-phase extraction [105,106], some adapted to high quantities of thorium [107]. Overall, despite the continuous qualification of new production sites in order to reach a sufficient supply of ^225^Ac for both preclinical and clinical use [108,109], effective and economic alternative production methods appear to be essential in view of the recent growing interest in ^225^Ac-based TAT [110].

Consequently, accelerator-based production techniques to obtain ^225^Ac have been developed. The most promising approach to obtain ^225^Ac at a large scale may be the cyclotron proton irradiation of a ^226^Ra target, involving the ^226^Ra(p,2n)^225^Ac transformation [111,112]. With a high cross-section peak (710 mb) at 16.8 MeV, this convenient method can be performed on low-energy cyclotrons. Moreover, it is not known to form either ^227^Ac (t_1/2_ = 21.8 y) or a significant amount of ^225^Ra (t_1/2_ = 14.9 d, obtained via the ^226^Ra(p,2n)^225^Ra reaction) byproducts. Short-half-life ^226^Ac (t_1/2_ = 29.4 h, obtained via the ^226^Ra(p,n)^226^Ac reaction) and ^224^Ac (t_1/2_ = 2.8 h, obtained via the ^226^Ra(p,3n)^224^Ac reaction) coproducts are formed, but their ratio with ^225^Ac decreases over time due to the differences in half-lives. Thus, the targets are processed 2–3 days after irradiation, dissolved in 0.01 M HCl and loaded on a Ln-spec column. Radium is washed through the column with 0.1 M HCl and ^225^Ac is eluted with 2 M HCl for a second purification on a Sr-spec column. Especially considering the high cross-section of this ^226^Ra target-based method [111,113], it could provide a valuable alternative for the large-scale and cost-effective production of ^225^Ac.

A second approach to obtain ^225^Ac via a cyclotron is the irradiation of a natural ^232^Th target with medium- to high-energy protons (>70 MeV), allowing the production of ^225^Ac through different pathways [104,105,106,114,115,116]. This reaction is characterized by a much lower cross-section than the ^226^Ra(p,2n)^225^Ac reaction and by the formation of several radioactive impurities such as the long-lived ^227^Ac (0.1 to 0.2%). Nevertheless, the low radioactivity and sufficient availability of ^232^Th allow easier target fabrication and processing [117,118,119].

Overall, it is likely that in order to meet the growing demand, the actinium used in preclinical and clinical applications will come from different production routes, which requires a harmonization of the quality criteria expected for this radioisotope.

### 3.2. Chemistry of Actinium

#### 3.2.1. Actinium in Aqueous Solution

Actinium is the chemical element with atomic number 89 and the first element of the actinide group, to which it gives its name. Nevertheless, actinium has rather similar chemical properties to lanthanum and other lanthanides. Actinium exists essentially in the +3 oxidation state in aqueous solution; additionally, Ac^3+^ is the largest +3 cation in the periodic table. It is also the most basic +3 ion due to its low charge density, directly related to its large size. Although the +3 state is the most stable in aqueous solution, the +2 oxidation state may also be encountered [120]. This second species is assumed because a reduction half-wave potential in a ^225^Ac^3+^ aqueous solution can be observed. The progressive negative shift of this potential in the presence of increasing 18-crown-6 concentrations has been attributed to the formation of a complex between crown ether and divalent actinium [121]. However, without the effect of 18-crown-6 on the reduction of ^225^Ac^3+^, the existence of stable ^225^Ac^2+^ ions in aqueous solution remains unlikely regarding the low extraction yields of actinium using sodium amalgam in aqueous sodium acetate, an extraction technique usually efficient for lanthanides at a stable +2 oxidation state [122]. Moreover, the reduction of Ac^3+^ to Ac^2+^ in aerobic aqueous conditions appears to be impossible according to theoretical studies that predicted markedly negative standard reduction potentials for these species (−4.9 V and −3.3 V/NHE, respectively) [123,124]. Due to the low charge density of this ion, Ac^3+^ hydrolysis is observed at slightly higher pH values than other Group 3 cations, such as La^3+^ or Y^3+^ [125,126,127]. The first hydrolysis constant (pK_1h_) of Ac^3+^ cations has been measured as 9.4 ± 0.1 (Table 1) [128]. This implies that pH values below this threshold are not in favor of water molecule coordination with Ac^3+^ followed by the release of a proton to form AcOH^2+^. Interestingly, this property may allow the consideration of a wide range of pH values for radiolabeling reactions with ^225^Ac, since for pH values lower than 9, inert hydrolyzed Ac ion formation is unlikely. Hence, in view of these properties, the coordination chemistry of the six-coordinate Ac^3+^ has been particularly studied to extend the applications of this radioelement in radiopharmacy and nuclear medicine.

Actinium, as well as other elements with an atomic number higher than lead (Z > 82), has no stable isotope, which makes chemical reactivity studies on this atom more challenging. Nevertheless, despite its slightly smaller ionic radius (1.03 Å vs. 1.065 Å) [129,130], the lanthanum cation La^3+^ has emerged as a convenient stable surrogate due to the comparable chemical properties between the Ac^3+^ ion and Ln^3+^ lanthanide ion [131,132]. Similarly, ^132^La, ^133^La and ^134^Ce have recently been suggested as PET-imaging surrogates for actinium-containing radiopharmaceuticals [133,134,135,136].

#### 3.2.2. Coordination Chemistry of Actinium

The usefulness of actinium-225 as a radionuclide for therapeutic purposes has been limited for a long time by the unavailability of chelating agents that are both capable of being compatible with this bulky radionuclide and of controlling the fate of the resulting daughter emitters, particularly with regard to their alpha-recoil, which is related to the conservation of momentum laws that occurs upon release of an alpha-particle [137]. Nevertheless, the coordination chemistry of such a clinically relevant alpha-emitter has recently gained more and more interest [138].

Considering its low polarizability and despite its large ionic radius, the Ac^3+^ ion is considered a hard Lewis acid [139], showing a medium absolute chemical hardness value of 14.4 eV [140]. As such, it will complex more easily with hard ligands, such as anionic oxygen donors. The complexation reaction will preferentially occur under charge control and the acid–base bond will be essentially ionic. Indeed, Ac^3+^ displays an electrostatic interaction constant (*E*_A_) value of 2.84 and a covalent interaction constant (*C*_A_) value of 0.28 [138]. This predominance of charge interactions can be predicted from the character of the frontier molecular orbitals, which are centered on the nuclei of the donor and acceptor atoms; when these atoms are close together in space, the overlaps of the orbitals are negligible while the charge interactions are strong. This is mainly attributed to the density of the charge, which is very significant in ions of hard consistency. Besides, the large ionic radius of the Ac^3+^ ion tends to induce the formation of kinetically unstable complexes. Thus, a wide variety of chelating agents have been studied for their coordination properties with actinium in order to identify complexes with both fast kinetic properties and high thermodynamic and in vivo stability.

**Figure 6:**
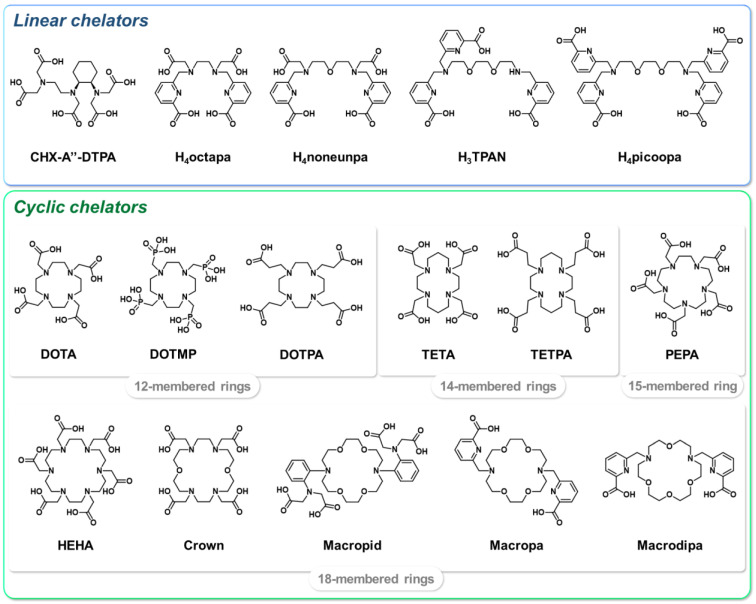
Selected chelators investigated for ^225^Ac complexation.

The high ionic radius of the Ac^3+^ cation suggests that the most suitable chelators would be polydentate agents, with a high denticity between 8 and 12. Initial works investigated the suitability of linear polyaminocarboxylate chelators, such as CHX-A″-DTPA, for the chelation of the ^225^Ac^3+^ cation [141,142]. These efforts were motivated by the advantageous radiolabeling kinetic properties of these ligands; however, the complexes obtained did not show sufficient in vivo stability. Subsequently, large macrocyclic chelators were considered and the 18-membered polyaminocarboxylic acid core HEHA was rapidly identified as particularly suitable for actinium complexation [142,143]. Nevertheless, once conjugated to vector molecules, HEHA formed insufficiently stable complexes with ^225^Ac, probably due to transchelation and radiolysis processes [144,145]. Other macrocyclic chelators were subsequently studied to address these drawbacks, but were still associated with either the instability of the complex (PEPA [141,142], DOTMP [146,147,148]) or low radiolabeling yields (TETA, TETPA, DOTPA [146], macropid [138,149]). Interestingly, the crown chelator, inspired by HEHA, displayed good ^225^Ac-chelating properties, both in its free form and when conjugated to a peptide [150,151]. Related diaza-18-crown-6 moieties H_2_macropa [152,153,154] and macrodipa [155,156,157], bearing picolinate arms, also formed stable complexes with large lanthanide ions such as ^225^Ac. In addition to a rapid complexation reaction at room temperature, ^225^Ac–macropa and macrodipa complexes remained stable both in vitro and in vivo. The interest in picolinic acid units as actinium chelators was also evidenced by acyclic derivatives such as octapa [158], H_4_noneunpa [159], H_4_picoopa [160] or H_3_TPAN [161], which have mostly been studied in vitro for their radiochemical properties up to now. Figure 6 summarizes the chemical structures of the ligands discussed herein.

Despite the large variety of chelators designed and studied to easily form stable complexes with actinium, the 12-membered macrocycle DOTA remains one of the reference chelating agents for this radioelement, especially for radiopharmaceuticals with clinical applications. More precisely, four-arm DOTA derivatives seem to be the most suitable for complexation with ^225^Ac [162]. Moreover, this chelator is already widely used in humans within theranostic radiopharmaceuticals such as DOTATOC and DOTATATE, which are already known to the regulatory authorities. Besides, the very versatile character of DOTA allows its stable coordination with various hard +3 radioactive ions such as Ga^3+^, In^3+^, Sc^3+^, Y^3+^, Lu^3+^ or even Tb^3+^ [163,164]. ^64^Cu-labeled DOTA bioconjugates are also currently used in the clinic, but have displayed slight transchelation effects and thus a lower in vivo stability over time [165]. With ^225^Ac, radiolabeling conditions to form a DOTA complex are generally conventional, requiring a buffer solution such as ammonium acetate to increase the pH from 5 to 7. Interestingly, ascorbic acid is very commonly added to the reaction medium or during formulation, in order to decrease the radiolysis effects caused by the ^225^Ac decay chain [162,166,167,168,169,170,171]. Radiolabeling reactions involving small molecules or peptides can be heated from 50 °C to 95 °C [158,172,173,174]. Harsher conditions have also been described, including microwave irradiation [172,175]. For heat-sensitive molecules, a two-step approach was reported involving the radiolabeling of the bifunctional chelating agent before bioconjugation [146]. However, this approach displayed a low radiochemical yield due to the degradation of the anchoring isothiocyanate moiety of the bifunctional agent. Optimized protocols based on the Michael addition reaction [176] or on click chemistry approaches [177,178] have been subsequently developed; nevertheless, one-step radiolabeling protocols with milder conditions are generally preferred and most frequently used [162,179]. Finally, most radiolabeling protocols are completed with a quenching step using a DTPA solution to capture the remaining ^225^Ac and free daughter radionuclides [172,180,181,182]. An overview of ^225^Ac radiolabeling conditions reported in the literature is provided in the Supplementary Materials. Noteworthily, the lack of details in the procedures of these radiolabeling reactions is significant, with some important information sometimes missing. The same applies to the stability studies of the ^225^Ac-labeled radioconjugates, although it has been shown that this property should be assessed for each individual radioconjugate [183].

Quality control (QC) is a key step in the production process of radiopharmaceuticals, and reliable methods, especially for radio-HPLC analysis, are still lacking for ^225^Ac-radiolabeled molecules [184]. The radiochemical purity of ^225^Ac-radiolabeled products is most often only determined by thin-layer chromatography (TLC), using either iTLC or silica-coated plates as the stationary phase and 0.05–0.5 M citrate buffer (pH 4–5) as the mobile phase. Under these conditions, free ^225^Ac^3+^ migrates with the solvent front (R_f_ = 1) while the ^225^Ac–ligand complex usually remains at the baseline (R_f_ = 0). Particular attention should be paid to the time frame in which the QCs are performed. Indeed, ^225^Ac can be quantified through the γ-emission of its daughter nuclides ^221^Fr and ^213^Bi, using 190–247 and 399–488 keV energy windows, respectively [185]. For this, radiochemical equilibrium with gamma-emitting daughter radionuclides must be reached (i.e., after >6.5 h) [186,187,188], as the ratio of ^225^Ac to ^221^Fr and ^213^Bi constantly changes before this point. However, to avoid delaying this QC and postponing the release of ^225^Ac-containing radiopharmaceutical preparations, analysis after 2 h has been validated as an accurate time point to assess the purity of the radiopharmaceutical [189]. Similarly, gamma-counting measurement protocols with no requirement of secular equilibrium between ^225^Ac and its daughter radionuclides have been developed [190]. Overall, special consideration should be given to the redaction of experimental protocols provided in scientific publications, as the values given for radiochemical purity or radiochemical yield are affected by the timing of the QCs.

For TAT agents used in clinical practice, the radiolabeling step can be performed by an industrial radiopharmaceutical laboratory due to the long half-life of ^225^Ac. Nevertheless, these radiopharmaceuticals are, to date, essentially prepared in-house by pioneer centers. In this context, the automated GMP-compliant production of ^225^Ac–DOTA radiopharmaceuticals is possible using cassette-based synthesis systems [191], which have widely spread with the rise of ^68^Ga radiochemistry [192]. This option would address radiation protection, regulatory and aseptic requirements, but would imply a costly and difficult implementation, with the need for expertise of the radiopharmacy team involved in the process. Such a particular approach has been exemplified with [^225^Ac]Ac–DOTATATE [193].

#### 3.2.3. Relevance of DOTA in Actinium Radiopharmaceuticals

The in vivo fate of the ^225^Ac–DOTA complex alone was initially shown to be safe, with only low activity amounts in liver and bone of BALB/c mice [142]. Subsequently, DOTA-bioconjugated constructs (either antibodies or peptides) also showed the sufficient stability of the complex, both in vitro [162,166,167,173] and in vivo [162,166]. Nevertheless, early studies raised some concerns about the compatibility of DOTA with actinium [142,146]. Indeed, the large ionic radius of the Ac^3+^ ion is not in favor of the good thermodynamic stability of the DOTA complex, which may also be subject to transmetalation with other cations. In order to minimize adverse in vivo effects associated with the loss of ^225^Ac and its daughter radionuclides (especially ^213^Bi, significantly increasing the kidney-absorbed dose [194]) from DOTA, several approaches have been considered, such as the co-administration of chelating agents or concomitant diuresis [195,196].

Overall, DOTA does not seem to be the most suitable chelator for ^225^Ac due to its coordination chemistry properties. Nonetheless, it remains to date the gold standard chelating agent for ^225^Ac radiolabeling in the clinic. Most importantly, the prior use in humans of the same DOTA-containing vector molecules radiolabeled by ^68^Ga or ^177^Lu, such as PSMA-617 or DOTATATE, from the regulatory authority perspective, encourages the accommodation of the flaws of DOTA for ^225^Ac to benefit from a more significant hindsight regarding the clinical use of the vector molecule. This is particularly the case for somatostatin analogs such as ^225^Ac-labeled DOTATATE, which was very swiftly used in a clinical setting.

### 3.3. Somatostatin Analogs Radiolabeled with ^225^Ac: Preclinical Studies

Only a few studies have reported preclinical efficacy results of ^225^Ac-radiolabeled somatostatin analogues, due to this group of vector molecules having already been widely studied with beta-emitters such as ^90^Y or ^177^Lu [197].

**Figure 7:**
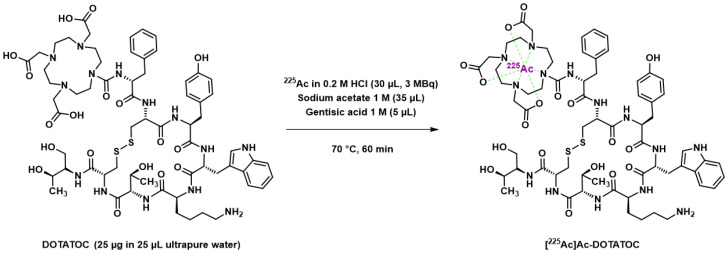
Radiolabeling reaction of DOTATATE with ^225^Ac according to Miederer et al. [198].

An initial study in 2008 explored the therapeutic efficacy of [^225^Ac]Ac–DOTATOC in nude mice bearing AR42J (rat pancreas neuroendocrine tumor) xenografts [198]. Radiolabeling reaction conditions involved sodium acetate buffer and gentisic acid as an anti-radiolytic compound (Figure 7); in addition, a rather moderate reaction temperature (70 °C, 60 min) allowed good radiochemical yields, probably facilitated by the large DOTATOC excess.

Activities between 10 and 60 kBq were well-tolerated by the mice; however, activities over 30 kBq induced pathologic changes in the renal cortex, suggesting radiation-induced acute tubular necrosis in both the distal and proximal tubules. Similar results were obtained in another study on Sprague Dawley rats that received 111 or 370 kBq [^225^Ac]Ac–DOTATOC and developed renal tubular nephrosis or renal glomerulopathy [199]. Only a slight accumulation in the liver was objectified, probably due to the release of free ^225^Ac. After a single administration of the highest non-toxic activity (20 kBq), tumor weights 14 days after treatment were lower with [^225^Ac]Ac–DOTATOC than with [^177^Lu]Lu–DOTATOC (1 MBq), in accordance with previous studies investigating [^213^Bi]Bi–DOTATOC [74,75]. This work therefore demonstrated the preclinical value of ^225^Ac-radiolabeled octreotide derivatives; nevertheless, some questions remained to be answered, such as their potential for chronic toxicity.

This point was addressed by Tafreshi et al. through a [^225^Ac]Ac–DOTATATE toxicity evaluation in BALB/c mice and an efficacy study in SCID mice bearing NCI-H69 or NCI-H727 (human small-cell and non-small-cell lung cancer, respectively) xenografts [200]. Interestingly, the reaction pH during radiolabeling was controlled with TRIS buffer, employed only for the ^225^Ac radiolabeling of other scarce compounds, either DOTA-conjugated antibodies [201] or peptides [166,173]. L-ascorbic acid was also preferred over gentisic acid. After a single injection of 55.5, 111 or 185 kBq in healthy mice, a 5-month follow-up highlighted a weight loss at ~100 days post-injection (p.i.) and a chronic progressive nephropathy for doses ≥111 kBq. Neither serum assays nor other organs showed pathologic changes related to treatment; [^225^Ac]Ac–DOTATATE thus appeared to have sufficient in vivo stability and tumor uptake, and its main toxicity was renal, as anticipated by analogy with its ^177^Lu analogues [202]. A single injection of ~145 kBq in xenografted mice led to a significant decrease in tumor volume 25 days p.i. prior to regrowth. This suggests the potential benefit of multiple injections in disease control. However, SSTR quantification showed the loss of some expression in regrowth tissues, implying the possible development of treatment resistance over time. Yet, the encouraging results obtained in these preclinical models leave room for [^225^Ac]Ac–DOTATATE as a new treatment for lung neuroendocrine neoplasms, as was the case with [^177^Lu]Lu–DOTATATE [203].

More recently, the radioconjugate [^225^Ac]Ac–macropa–octreotate (macropatate) was studied both in vitro and in vivo, with regard to the excellent ^225^Ac-chelating properties of the complexing agent macropa [204]. In addition to allowing milder complexation reaction conditions (RT for 1 h vs. 70 °C for 1 h), macropatate showed a comparable affinity for DOTATATE (21 nM vs. 22 nM on U2OS-SSTR2 cells) and, importantly, a slightly better in vitro serum stability (98% vs. 95% after 10 days). [^225^Ac]Ac–macropatate evaluation on a NCI-H69 xenografted mouse model (46.3 kBq, single injection) demonstrated the potential of this radioconjugate to delay tumor growth and improve survival; however, the results obtained with the [^177^Lu]Lu–DOTATATE comparator proved to be better (55-day survival vs. >100 days for 80% mice). Moreover, after an initial reduction in volume, tumors treated with [^225^Ac]Ac–macropatate subsequently relapsed, while tumors treated with [^177^Lu]Lu–DOTATATE showed durable remission. Interestingly, liver and kidney uptakes were higher than [^225^Ac]Ac–DOTATATE, which was explained by the authors as a result of the greater lipophilicity of macropatate, slowing renal clearance and increasing liver uptake. In summary, this study calls for optimization of the macropatate construct to achieve better in vivo properties, leaving the door open for the preclinical evaluation of other new bioconjugates with innovative ^225^Ac-chelating agents.

## 4. Clinical Use of ^225^Ac–DOTATATE

To date, [^177^Lu]Lu–DOTATATE is considered as the standard PRRT treatment for GEP NETs. In this regard, the phase 3 randomized control trial NETTER-1 specifically demonstrated that [^177^Lu]Lu–DOTATATE therapy plus long-acting octreotide was associated with a significantly longer PFS (28.4 vs. 8.5 months) than high-dose long-acting octreotide in advanced midgut GEP NET patients, although the OS endpoint of this study did not reach statistical significance (48 vs. 36.3 months, *p* = 0.3) [62,205]. Thus, this therapy offers a promising option as an early-line treatment for advanced NET [60,62]. Nevertheless, this type of pathology is known to frequently relapse, which may lead to patient retreatment. In this context, several studies have investigated the value of a renewed treatment with β-PRRT; furthermore, TAT protocols using somatostatin analogs, especially ^225^Ac-based approaches, were also rapidly proposed as an alternative for patients that did not respond to β-PRRT.

Although it was used several years earlier, the first literature report of an alpha-PRRT with [^225^Ac]Ac–DOTATOC in human dates from October 2018 [206]. Ten patients with metastatic NETs progressing after ^90^Y– and/or [^177^Lu]Lu–DOTATOC therapy were treated with intra-arterial [^225^Ac]Ac–DOTATOC (~8 MBq). Overall, the treatment was well-tolerated and effective, demonstrating its potential as a possible therapeutic alternative in advanced NETs resistant to β-PRRT.

Then, two major studies involving ^225^Ac-labeled octreotide analogs were reported in patients with advanced-stage SSTR-expressing metastatic GEP NETs. These works primarily focused on the hematologic and renal toxicity of [^225^Ac]Ac–DOTATOC, and on the long-term outcomes of this therapeutic, respectively.

### 4.1. Early Retrospective Study, in Search of the Best Regimen

Kratochwil et al. firstly reported, through a retrospective cohort study, the use of [^225^Ac]Ac–DOTATOC as an experimental salvage therapy in patients with aggressive, late-stage, or β-PRRT-resistant tumors [207]. In addition to gathering preliminary efficacy data, this work investigated the most appropriate treatment regimen and maximum cumulative dose of [^225^Ac]Ac–DOTATOC, especially with regard to hematologic and renal toxicity. Each of the 39 patients of this study (mean age: 58 (17–85) years old) expressed SSTRs according to [^68^Ga]Ga–DOTATOC PET/CT imaging (higher than liver background; Krenning score >2). The histological diagnosis of the 39 tumors is detailed in Table 2. All patients were ineligible or had already exhausted the approved treatments and received at least one cycle of [^225^Ac]Ac–DOTATOC therapy, from July 2011 to March 2015. Predictably, 37 patients (95%) were already pretreated, of which 82% were with β- (27/39) and/or α-PRRT (5/39) (Table 2).

**Figure 8:**
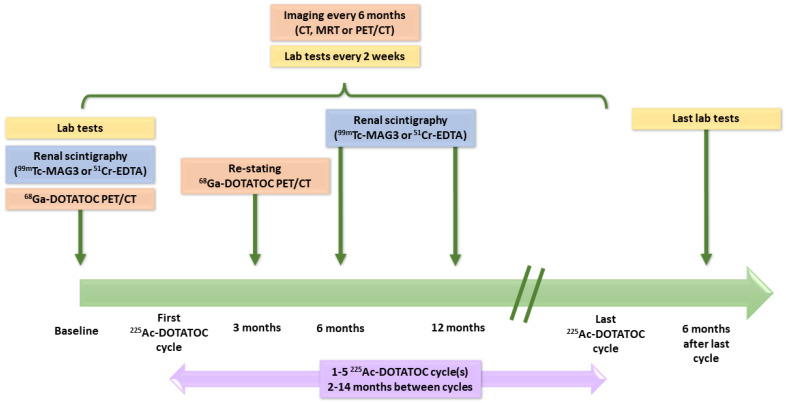
Course of the retrospective study reported by Kratochwil et al. [207].

Among the 32 patients pretreated with β- or α-PRRT, some received several radionuclides before ^225^Ac (Table 3). Hematologic and renal toxicities, as well as treatment efficacy, were monitored over time (Figure 8). CTCAE criteria were used to qualify hematologic toxicity, whereas estimates of the glomerular filtration rate (eGFR) were calculated from plasma creatinine values using the MDRD formula. Regarding the administrations of [^225^Ac]Ac–DOTATOC, it is essential to consider that, because of its pioneering use, both the activities injected and the intervals between administrations were determined consensually on a case-by-case basis, considering several individual criteria such as the tumor burden, sites of metastases or general clinical condition of the patient. Thus, this study brought together patients with a wide variety of treatment plans.

The evaluation of acute hematologic toxicity in all 39 patients highlighted a dose-dependent effect for thrombocytopenia and leukopenia. For single-dose administration, an activity below 44 MBq was associated with grade 0 to 2 hematologic toxicity. In contrast, for a dose above 45 MBq and above 60 MBq, grade 3 and 4 adverse events were recorded, respectively. Among the 39 patients included in this study, 24 had a second cycle, 6 had a third cycle, 2 had a fourth cycle and 1 had a fifth cycle, allowing for a myelotoxicity evaluation after multiple doses. With repeated administrations, additive toxicity was observed if the subsequent doses were not reduced or for short intervals between cycles. Indeed, seven of the eight grade 3–4 hematologic adverse events recorded in this study occurred with succeeding cycles of >25 MBq [^225^Ac]Ac–DOTATOC, which suggested a dose-dependent toxicity. Thus, the hematologic toxicity could be reduced by setting a maximum dose of ~44 MBq for single-dose regimens and by adjusting both the further doses (from 20 to 25 MBq) and the intervals between cycles (optimum interval of 4 months) for repeated treatment regimens.

**Figure 9:**
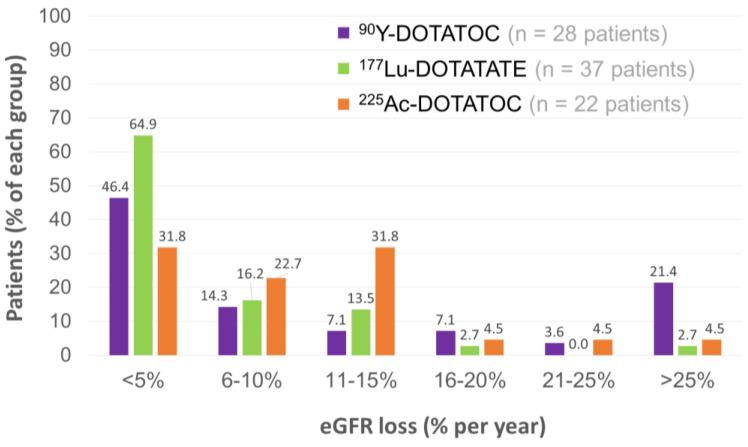
Distributions of patients with respect to the extent of annual GFR loss for β-PRRT and TAT [207,208,209,210].

Renal toxicity was evaluable in 22 patients, with a median follow-up of 57 (18–90) months. Chronic kidney disease (CKD) was the most relevant and late effect. However, pre-existing risk factors for CKD were found in 36/39 patients. In addition, most of them (32/39) had already received β-PRRT that probably induced a lower kidney tolerance. A mean decrease of 7.6% and 14% in tubular excretion rate values were observed in the first 6 months and the first 18 months, respectively. The severity of eGFR losses was further studied and compared with previous data of patients treated with β-PRRT [208,209,210]. A higher fraction of 6–10% and 11–15% eGFR loss per year was observed with TAT, versus β-PRRT (Figure 9). Conversely, [^225^Ac]Ac–DOTATOC appeared to cause substantially less eGFR loss (<5% per year) than [^177^Lu]Lu–DOTATATE. Nevertheless, the susceptibility of these different groups of patients to the renal toxicities of PRRT was not strictly comparable considering their respective previous treatments.

Overall, this early clinical experience with [^225^Ac]Ac–DOTATOC suggested that this strategy could provide a clinical benefit in selected patients not responding to β-PRRT or with poor tumor prognosis. The toxicity profile of [^225^Ac]Ac–DOTATOC appeared to be manageable if adequate intervals between cycles and reasonable doses were planned, typically ~20 MBq per cycle every 4 months for a cumulative dose up to 60–80 MBq.

### 4.2. Subsequent Evaluation in Patients Revealing New Clinical Outcomes

After reporting the efficacy and safety of [^225^Ac]Ac–DOTATATE in nine patients with paraganglioma [211], Ballal et al. conducted a study presented as prospective, involving a cohort of 91 well-differentiated inoperable or metastatic SSTR-expressing GEP NET patients in order to explore the long-term outcomes of [^225^Ac]Ac–DOTATATE [212]. The preliminary data have been published since April 2018 [213,214,215,216,217,218]. In the final report of this study, patients were categorized into three groups depending on their pretreatment with ^177^Lu-PRRT: prior ^177^Lu-PRRT refractory group (33 patients), prior ^177^Lu-PRRT disease control group (24 patients) and ^177^Lu-PRRT naïve group (34 patients). The mean age of this cohort was 54 (25–75) years old. Pancreatic NETs accounted for 33%, followed by duodenum and ileum NETs (14.3% and 13%). The majority of patients (81/91) had a grade 1/2 disease according to the WHO classification of GEP NETs (Table 4) and all patients were metastatic according to [^68^Ga]Ga–DOTANOC PET/CT (96.7%, 72.5% and 27.5% patients had liver, lymph node and bone metastases, respectively). Table 4 specifies the previous treatment received by the overall population; 10 patients were still on long-acting somatostatin analogs, which were stopped 4 weeks before starting [^225^Ac]Ac–DOTATATE. 

**Figure 10:**
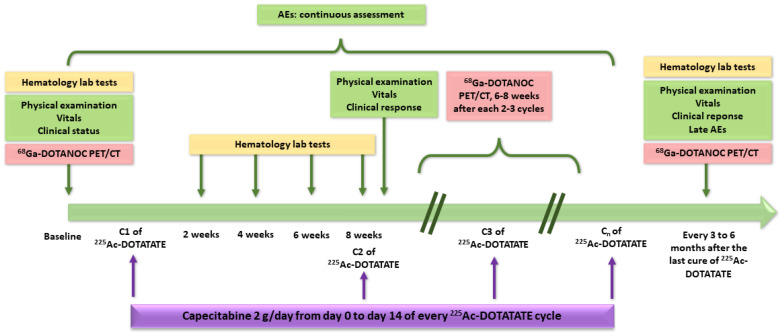
Design of the study reported by Ballal et al. [212]. Clinical status and clinical response were assessed with Karnofsky performance status and Eastern Cooperative Oncology Group (ECOG) performance status. AEs: adverse events.

The study regimen of each group consisted of 100 to 120 kBq/kg body weight of [^225^Ac]Ac–DOTATATE (i.e., activities approximately two times lower than those reported by Kratochwil et al.) administered in two-month intervals. Capecitabine was given to all patients (1 g twice a day from day 0 to day 14 of every [^225^Ac]Ac–DOTATATE cycle) as a radiosensitizer [219]. Figure 10 summarizes the design of the study. 

Cumulatively, 453 cycles were administered. The mean cumulative dose of [^225^Ac]Ac–DOTATATE was 35.52 (21.64–59.47) MBq and the median time interval between two cycles was 8 weeks from April 2018 to October 2021. Three patients (3.3%) received 1 cycle of [^225^Ac]Ac–DOTATATE, 29 patients (31.9%) received 2 to 3 cycles and 59 patients (64.8% received 4 to 10 cycles. The median number of cycles per patient was 4. Table 5 details these data and illustrates the heterogeneity in the duration of TAT management in this cohort.

After a median follow-up duration of 24 (5–41) months, the median OS was not attained at the time of analysis; thus, a 24-month survival probability of 70.8% was calculated. Upon univariate analysis, the presence of bone metastases, a cumulative dose of ^225^Ac–DOTATATE < 37 MBq and a progression of disease (PD) with ^225^Ac–DOTATATE were associated with significantly poorer OS (*p* < 0.030, *p* < 0.0003 and *p* < 0.0001, respectively). Concerning PFS, its median was not reached in the overall patient population either. Upon univariate analysis, as for OS, the presence of bone metastases, a cumulative dose of ^225^Ac–DOTATATE < 37 MBq and PD with ^225^Ac–DOTATATE were associated with a significantly reduced PFS (*p* < 0.028, *p* < 0.028 and *p* < 0.0009, respectively).

Objective tumor response (or morphological response) according to RECIST 1.1 criteria (for primary site, node and viscera) 6–8 weeks after completing every 2–3 cycles of ^225^Ac–DOTATATE was evaluated in the three patient groups. Only 2 of the 79 evaluable patients, both previously pretreated with ^177^Lu-PRRT, achieved a complete response (CR), whereas no CR was observed in the naïve ^177^Lu-PRRT group. There were 38 partial responses (PRs), 23 stable diseases (SDs) and 16 PDs. Table 6 details the morphological responses obtained according to the pretreatment with ^177^Lu-PRRT and the disease status at the time of recruitment.

For patients still alive at the end of analysis, there was a significant improvement in clinical performance status from baseline to the end of analysis: the median Karnofsky performance status increased from 60 to 70 (*p* < 0.0001) and the median ECOG score changed from 2 to 1 (*p* < 0.0001). These improvements were not significant in the overall population. 

Regarding the tolerance of [^225^Ac]Ac–DOTATATE, none of the patients in the entire series encountered grade 4–5 hematologic or renal toxicities according to CTCAE v5.0 criteria. Only one patient experienced grade 3 thrombocytopenia, which suggests that [^225^Ac]Ac–DOTATATE safety could be comparable to [^177^Lu]Lu–DOTATATE in the NETTER-1 clinical trial. In future long-term safety studies, it may be relevant to investigate the late incidence of myelodysplastic syndrome in patients treated with TAT to compare with β-PRRT. In [^225^Ac]Ac–DOTATATE-treated patients, grade 1/2 hematologic toxicities were mostly prevalent at baseline. Grade 1/2/3 adverse events were reported at the end of the assessment (fatigue, loss of appetite, nausea, gastritis, abdominal pain and distension, myalgia and flushing) but were also prevalent at baseline. Importantly, 10 malignant ascites and 1 grade V pleural effusion were reported. In view of these outcomes, Ballal et al. concluded a limited and manageable toxicity of [^225^Ac]Ac–DOTATATE for doses of 100–120 kBq/kg body weight with a treatment interval of two months. Moreover, this study demonstrated that the resensitization of a [^177^Lu]Lu–DOTATATE refractory tumor was possible (CR for 1/33, PR for 7/33 and SD for 11/33 patients). Nevertheless, the design of this study can be questioned; in particular, Strosberg et al. raised several important points for discussion [220]. Indeed, the authors described this study as prospective, but the sample size was not predetermined, strict inclusion criteria and interpretation of responses were missing, and no clear prospective treatment protocol was defined. Furthermore, the eligibility criteria changed between the short-term analysis of the study [214] and the most recent report [212]. Another important point is that the co-administration of capecitabine was not mentioned in the initial report. With regard to these limitations, the authors pointed out that the study of such a heterogeneous population allowed the inclusion of poor-outcome patients and thus better represented real world clinical settings.

Overall, the best outcome was reached by patients who achieved disease control (either PR or SD) with prior ^177^Lu-PRRT before being retreated with [^225^Ac]Ac–DOTATATE (24-month OS probability = 95% vs. 55.6% and 62.6% in the ^177^Lu-PRRT refractory and naïve groups, respectively). This result calls for further investigations to better define the place of ^225^Ac-TAT in the comprehensive treatment strategy for patients with advanced metastatic NETs, potentially as a PRRT retreatment option in a salvage setting.

### 4.3. Relevance and Benefits of Retreatment in Patients Managed with PRRT

Van der Zwan et al. evaluated ^177^Lu-PRRT salvage therapy in a large retrospective cohort of patients with progressive bronchial NETs or GEP NETs who had benefitted from initial PRRT (I-PRRT) with a minimal PFS of 18 months [221]. For the salvage group, 168 patients received two more cycles of PRRT (R-PRRT group) and 13 patients received a second retreatment of two more cycles (RR-PRRT group). A non-randomized control group of 99 patients only received I-PRRT. The overall median follow-up time was 88.6 months from the start of I-PRRT. Table 7 specifies the median cumulative doses over the salvage and control group after I-PRRT, R-PRRT and RR-PRRT. R-PRRT resulted in 15.5% ORR and 59.5% SD at 3 months. RR-PRRT resulted in an ORR of 38.5% and SD of 53.8%. Radiological tumor responses after I-PRRT and R-PRRT were significantly correlated (*p* < 0.01), as well as PFS after I-PRRT and after R-PRRT (*p* < 0.01). Concerning OS, the salvage group had a significantly longer OS than the non-randomized control patients (*p* < 0.01): 80.8 months vs. 51.4 months. Toxicities were similar in the salvage and control groups. No grade 3–4 nephrotoxicity occurred, and hematologic toxicity was similar in the two groups, which is quite consistent with the results gathered from the cohorts of patients treated with ^225^Ac salvage PRRT.

Similarly, a retrospective study reported by Sabet et al. introduced the feasibility of retreating GEP NET patients with [^177^Lu]Lu–DOTATATE in cases of initial response to this PRRT [222]. Thirty-three patients (14/33 pancreatic NET and 19/33 non-pancreatic NET) received a median of 2 (1–4) cycles of salvage therapy with a mean administered activity of 17.7 (8.0–33.2) GBq and a cumulative activity of 44.3 (30.0–83.7) GBq. Retreatment with ^177^Lu-PRRT resulted in 24.2% objective radiological responses (ORRs) and 42.4% SDs. The median PFS was 13 (9–18) months from the start of the salvage therapy and 22 (19–25) months after the initial ^177^Lu-PRRT. Remarkably, these results are slightly lower than those obtained in the pretreated subgroups of the Ballal et al. study involving [^225^Ac]Ac–DOTATATE [212]. It is also interesting to note that patients with a durable PFS after the initial ^177^Lu-PRRT tended to have a longer PFS after salvage ^177^Lu-PRRT (*p* = 0.04), possibly because of less aggressive diseases. In this study, hematologic toxicity was considerably higher after the salvage PRRT compared to the data reported in previous studies after standard treatment with [^177^Lu]Lu–DOTATATE (cumulative activity < 29.6 GBq) [51]. Indeed, high cumulative activities (30.0–83.7 GBq) led to relevant grade 3–4 hematologic toxicity in 16.5% of administrations and in 21.2% of patients. However, this hematologic toxicity was considered acceptable because all patients returned to normal blood cell counts and no myelodysplastic syndrome was observed. Moreover, no grade 3–4 nephrotoxicity was noticed. 

A study of the same kind was conducted by Rudisile et al. on 35 patients objectified a median PFS after an initial PRRT of 33 months (95% CI 30–36) and a median OS not reached by 25 months after the start of salvage PRRT [223]. Similarly, Vaughan et al. focused on the retreatment with either ^90^Y– or ^177^Lu–DOTATATE of 47 patients, the majority of which had previously been treated with ^90^Y–DOTATATE [224]. The median PFS after retreatment was 17.5 months (95% CI 11–23.8) with no significant difference depending on the radiopharmaceutical.

Yordonova et al. conducted a retrospective study in a cohort of 15 patients, with a mean age of 58 years old, to assess the safety of repeated ^177^Lu-PRRT in patients with recurrent NETs [225]. Patients had either a pancreatic (7/15), midgut (3/15), gastric (1/15), renal (1/15) or an unknown primary disease (3/15). Before baseline therapy with ^177^Lu-PRRT, patients were pretreated with surgery (6/15), biotherapy (7/15), chemotherapy (2/15) and/or ^90^Y-PRRT (1/15). Each patient received a median of 9 (8–13) cycles, which included the baseline therapy (median 4 (3–7) cycles) and the salvage therapies (overall median 5 (3–9) cycles). The median administered activity was 63.9 (52–96.6) GBq. Noteworthily, a decrease in PFS was observed with additional salvage treatments. This can be explained either by an increase in the tumor aggressiveness or by a decrease in the radiation sensitivity of the tumor. However, a significant prolongation of the mean OS (85.6 months for retreated patients vs. 69.7 months for patients after only baseline PRRT, *p* < 0.001) was highlighted. Concerning tolerance, none of the patients showed grade 3–4 nephrotoxicity; the predominant hematologic toxicity was leukopenia (2/15 patients had grade 3). Generally, more toxicities occurred during the baseline therapy (grade 3 leukopenia and gastrointestinal bleeding, 23%) than during salvage therapy (grade 3 leukopenia and abdominal pain, 13%).

These encouraging results based on retrospective studies of salvage PRRT with ^177^Lu–somatostatin analogues have led to an ongoing prospective multicenter randomized clinical trial (ReLUTH clinical trial, NCT04954820). This trial will assess the schemas of retreatment comparing two vs. four cycles in a population of patients with well-differentiated midgut neuroendocrine tumors presenting with a new progression after the first course of ^177^Lu–DOTATATE. The primary endpoint is defined as the disease control rate at 6 months after randomization, and safety will be evaluated as one of the secondary endpoints [226]. 

Overall, it appears that the outcomes from studies involving retreatment with β-PRRT vary substantially between reports, probably due to different individual and tumor factors. In order to more clearly define the place of ^225^Ac-PRRT in such retreatment strategies, prospective comparative studies comparing β- and α-PRRT retreatment could provide crucial information on the most appropriate use of these two approaches.

### 4.4. Case Reports of ^225^Ac–DOTATATE Clinical Use

**Figure 11:**
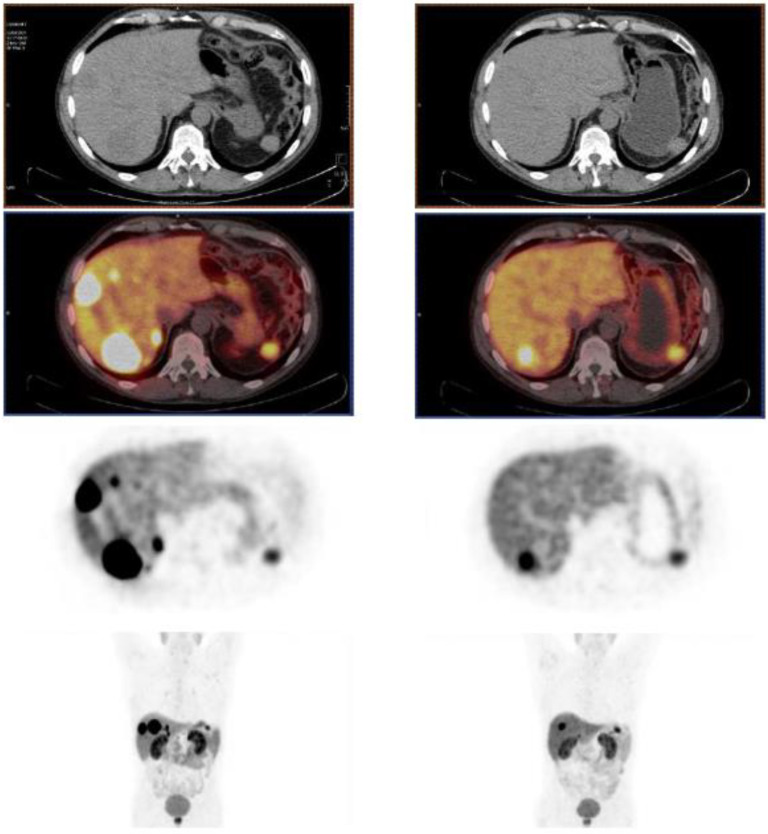
[^68^Ga]Ga–DOTATATE PET/CT in the patient described by Budlewski et al. [227], showing important uptake in hepatic NET metastases after treatment with a long-acting somatostatin analog plus everolimus (**left**), and significant decrease in lesion uptake after two cycles of [^225^Ac]Ac–DOTATATE associated with good metabolic and structural response (**right**).

Aside from the previously presented cohort studies with ^225^Ac-labeled somatostatin analogs, a few case reports also exemplify the good tolerance and effectiveness of ^225^Ac-based TAT targeting SSTRs. For instance, a 76-year-old patient with a well-differentiated, functional pancreatic NET with hepatic metastases achieved PR according to RECIST 1.1 criteria after a single 9.8 MBq administration of [^225^Ac]Ac–DOTATOC [227]. The tolerance was good despite pretreatment with 10 cycles of β-PRRT (cumulative dose of 57.8 GBq of ^177^Lu/^90^Y). An improvement of clinical symptoms and good tolerance were also demonstrated in a 70-year-old patient with a metastatic, well-differentiated pancreatic NET after one cycle of 7 MBq [^225^Ac]Ac–DOTATATE [87]. No additional toxicity was observed despite a prior cumulated dose of 48 GBq of [^177^Lu]Lu–DOTATATE. Interestingly, several reports have documented the treatment of NETs with α-PRRT with no previous use of β-PRRT. Budlewski et al. described the case a 66-year-old patient with a metastatic pancreatic NET in therapeutic failure with cold somatostatin analogs and everolimus [228]. As illustrated in Figure 11, the administration of 16.4 and 14.3 MBq [^225^Ac]Ac–DOTATATE at 8-week intervals resulted in good disease control (monitored by PET imaging and serum chromogranin A dosing) and good tolerance. 

**Figure 12:**
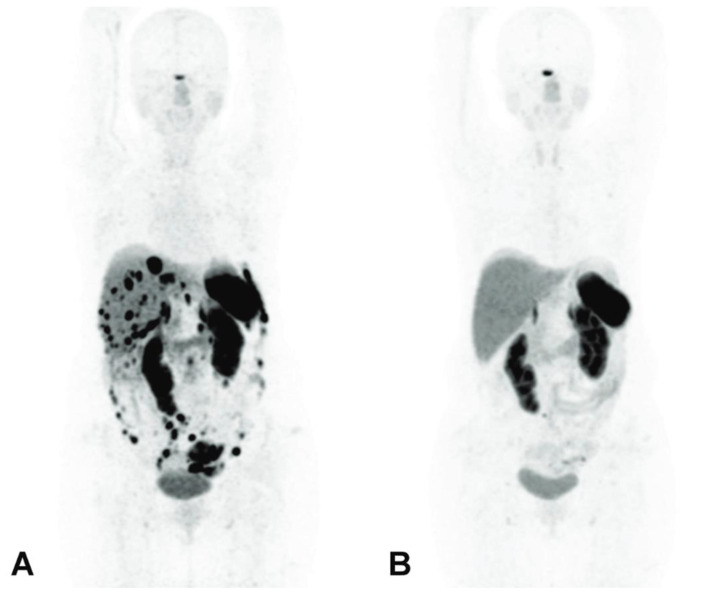
[^68^Ga]Ga–DOTATATE PET/CT imaging before α-PRRT (**A**) showing >50 SSTR-positive abdominal lesions, and 3 months after a single cycle of 10 MBq [^225^Ac]Ac–DOTATATE (**B**) showing the disappearance of all the abdominal lesions with the exception of a 5 mm lymph node in the para-aortic region [228].

In a 72-year-old patient diagnosed with a grade 2 NET, 5.5 MBq of first-line [^225^Ac]Ac–DOTATATE was also considered effective, with a particular emphasis placed on the relevance of post-therapy SPECT/CT imaging to track the biodistribution of the tracer and for dosimetry purposes [82]. Similarly, a 46-year-old woman with a metastatic grade 2 and heavily pretreated NET with numerous lesions in the abdomen, liver and peritoneal space achieved a spectacular and almost complete response after a single administration of 10 MBq [^225^Ac]Ac–DOTATATE [229] (Figure 12).

In a 46-year-old patient with a multi-metastatic rectal NET associated with lytic and sclerotic bone lesions, first-line TAT was preferred to β-PRRT. A partial morphological and molecular response was achieved after six cycles of [^225^Ac]Ac–DOTATATE (100 kBq/kg) with a complete resolution of symptoms. The patient was clinically stable 6 months after treatment [230]. Finally, a thyroid dysfunction was reported in a 55-year-old patient with well-differentiated metastatic NET, one month after the last of four cycles of [^225^Ac]Ac–DOTATATE [231]. Thyroid function values returned to normal within 6 months, suggesting subclinical hypothyroidism. This may be explained by the destruction or inflammation of the thyroid gland in the acute phase after TAT, followed by fibrosis in the chronic phase [232].

It is interesting to point out the high heterogeneity of the administered doses in these individual reports that may vary by twice as much. This illustrates the need to formally define an optimal treatment regimen and to investigate the most relevant criteria for dose adjustment.

## 5. Conclusions and Perspectives

Ahead of other α-emitters, TAT using ^225^Ac-labeled somatostatin analogs seems to be a promising therapeutic approach for metastatic or inoperable NETs, especially considering its preliminary efficacy and safety results. Efforts to achieve the sufficient production of ^225^Ac and extensive radiochemistry works aimed at optimizing the chelation of this radioelement reflect the high expectations for its clinical use, including in other pathologies such as prostate cancer with ^225^Ac-labeled PSMA ligands [88,233,234,235,236,237], or even in hematological cancers such as acute myeloid leukemia [238]. However, the role of TAT versus β-PRRT in terms of OS, PFS and long-term toxicity is still difficult to define without formal comparative studies. Beforehand, the further investigation into the therapeutic use modalities of ^225^Ac-radiolabeled somatostatin analogs will be required. Some of these questions may be answered by the ACTION-1 clinical trial (NCT05477576) [239], which is designed to determine the safety, pharmacokinetics, and recommended phase 3 dose of [^225^Ac]Ac–DOTATATE and its efficacy compared to the investigator-selected standard of care therapy in patients with inoperable GEP NETs that progressed following ^177^Lu–somatostatin analogues. Similarly, preliminary data on the efficacy of ^225^Ac-labeled somatostatin analogs in other cancers such as paragangliomas [211] or pheochromocytomas [240] will need to be further consolidated. From a radiopharmaceutical perspective, the importance of developing a reliable method for measuring the radiochemical purity of ^225^Ac conjugates produced in-house appears to be crucial and would certainly be a key requirement to obtain approval for clinical use from regulatory agencies. In addition, it will be interesting to develop a standardized dosimetric tool for the accurate estimation of adsorbed doses in target and non-target organs. For the time being, TATs constitute an emerging therapeutic alternative for patients with either highly resistant or late-stage disease, particularly in the context of compassionate access, depending on the country. 

## Figures and Tables

**Table 1:** Chemical and structural properties of the actinium nucleus and the Ac^3+^ ion.

Property	
Atomic configuration	5f^0^ 6d^1^ 7s^2^
Oxidation states (in acid non-complexing aqueous solution)	3
Metallic radius (Ac^0^)	1.88 Å
Six-coordinate ionic radius (Ac^3+^)	1.065 Å
pK_1h_	9.4
Absolute chemical hardness (*η*)	14.4 Ev
Electrostatic contribution in complexes formation (*E*_A_)	2.84
Covalent contribution in complexes formation (*C*_A_)	0.28
Ionicity (I_A_ = *E*_A_/*C*_A_)	10.14
Hydration number	10.9 ± 0.5
Ac–O(H_2_O) bond length	2.59 to 2.63 Å

**Table 2:** Histological diagnosis of the 39 patients reported by Kratochwil et al. and details of the prior systemic treatments for the 37 pretreated patients [207].

		Number of Patients (%)
**Histological diagnosis**	GEP NET	22/39 (56.4%)
	Lung carcinoid NET	5/39 (12.8%)
	Lung NEC	1/39 (2.55%)
	Unknown primary NET	4/39 (10.3%)
	Medullary thyroid carcinoma	2/39 (5.2%)
	Meningioma (WHO II)	1/39 (2.55%)
	Merkel-cell carcinoma	1/39 (2.55%)
	Paraganglioma	1/39 (2.55%)
	Prostate NET	1/39 (2.55%)
	Renal NET	1/39 (2.55%)
**Previous therapy**	β- or α-PRRT	32/39 (82.0%)
	SSA	21/39 (53.8%)
	Chemotherapy	16/39 (41.0%)
	TKI	8/39 (20.5%)
	SIRT	4/39 (10.3%)
	TACE	1/39 (2.6%)
	Immunotherapy	2/39 (5.1%)
	Interferon	2/39 (5.1%)

SSA: somatostatin analogue; TKI: tyrosine kinase inhibitor; SIRT: selective internal radiotherapy; TACE: trans-arterial chemoembolization.

**Table 3:** Details of PRRT for the 32 patients pretreated.

Radionuclide	Number of Patients	Mean Cumulative Dose in GBq (Min–Max)	Median Cumulative Dose in GBq
^90^Y	25	9.2 (2–22)	8
^177^Lu	29	12.7 (1–44)	12
^213^Bi	5	11 (4–19)	13

**Table 4:** Site of primary tumor, WHO grades and prior treatments received by the patients involved in the study of Ballal et al. [212].

Primary Tumor Site	Number of Patients (%)
Pancreas	30 (33%)
Stomach	7 (7.7%)
Appendix	1 (1%)
Ileum	12 (13%)
Duodenum	13 (14.3%)
Jejunum	2 (2.2%)
Colon	2 (2.2%)
Rectum	8 (8.8%)
Abdominal with unknown primary	16 (17.6%)
**WHO Tumor grade (Ki67 index)**	
Grade 1 (<2%)	33 (36.2%)
Grade 2 (3–20%)	48 (52.7%)
Grade 3 (>20%)	7 (7%)
**Prior treatment before ^225^Ac–DOTATATE**	
Surgery	21 (23%)
SSA	70 (77%)
Chemotherapy (cytotoxic or TKI)	18 (20%)
[^177^Lu]Lu–DOTATATE	57 (62.6%)

SSA: somatostatin and somatostatin analogues; TKI: tyrosine kinase inhibitor.

**Table 5:** Number of [^225^Ac]Ac–DOTATATE cycles assessed by patients.

Number of Cycles with 2 Monthly Intervals at the Time of Analysis	Number of Patients (%)
1	3/91 (3.2%)
2	13/91 (14.3%)
3	16/91 (17.6%)
4	15/91 (16.5%)
5	7/91 (7.7%)
6	11/91 (12.1%)
7	9/91 (9.9%)
8	5/91 (5.5%)
9	8/91 (8.8%)
10	4/91 (4.4%)

**Table 6:** Treatment details and response in the three groups of patients post cycles of ^225^Ac–DOTATATE therapy.

	Prior ^177^Lu-PRRT (n = 57)		^177^Lu-PRRT Naïve (n = 34)
	DP (n = 33)	SD/PR (n = 24)	
Mean cumulative activity of ^177^Lu-PRRT	25.7 ± 12.7 GBq (5.5–49.5)	25.6 ± 10 GBq (7.4–39)	-
Mean cumulative activity of ^225^Ac-PRRT	39.6 ± 24.2 MBq (12–100)	48.6 ± 19.4 MBq (8.9–80)	35 ± 20 MBq (6–77.7)
CR	1 (3.03%)	1 (4.17%)	0
PR	7 (21.21%)	16 (66.66%)	15 (44.12%)
SD	11 (33.33%)	5 (20.83%)	7 (20.59%)
PD	11 (33.33%)	1 (4.17%)	4 (11.76%)
Not assessed	3 (9.10%)	1 (4.17%)	8 (23.53%)
24-month OS probability	55.6%	95.0%	62.6%
Alive	17 (51.5%)	21 (87.5%)	27 (79.4%)
Dead	16 (48.5%)	3 (12.5%)	7 (20.6%)
DSD	10	0	4

NA: not applicable; DSD: disease-specific death.

**Table 7:** Overall results presented in the retrospective study of Van der Zwan et al. [221].

	Control Group	Salvage Group		
	I-PRRT (n = 99)	I-PRRT (n = 168)	R-PRRT (n = 168)	RR-PRRT (n = 13)
Median cumulative dose (GBq)	29.9 GBq (18.6–30.7)	29.8 GBq (21.8–30.6)	14.9 GBq (3.7–16.2)	15.0 GBq (14.7–15.3)
Median cumulative administered dose	-	-	44.7 GBq (26.3–46.4)	59.7 GBq (55.2–60.5)
Response				
CR	-	1 (0.6%)	-	-
PR	36 (36.4%)	93 (55.4%)	26 (15.5%)	5 (38.5%)
SD	58 (58.6%)	73 (43.5%)	100 (59.5%)	7 (53.8%)
PD	-	-	33 (19.6%)	1 (7.7%)
Clinical PD	-	-	3 (1.8%)	-
Not evaluable	5 (5.1%)	1 (0.6%)	1 (0.6%)	-
Response at 3 months follow-up				
CR	-	-	-	-
PR	-	-	14 (8.3%)	2 (15.4%)
SD	-	-	111 (66.1%)	9 (69.2%)
PD	-	-	34 (20.2%)	2 (15.4%)
Clinical PD	-	-	3 (1.8%)	-
Not evaluable	-	-	1 (0.6%)	-
Died before the start of follow-up	-	-	5 (3.0%)	-
Median PFS			14.6 months (12.4–16.9)	14.2 months (9.8–18.5)
OS	51.4 months (46.7–56.1)	80.8 months (66.0–95.6)		

## Data Availability

The data presented in this study are available in the article and supplementary material.

## Supplementary Materials

The following supporting information can be downloaded at: https://www.mdpi.com/article/10.3390/pharmaceutics15041051/s1, Table S1: Selected ^225^Ac radiolabeling reaction conditions and quality control procedures found in scientific literature.
